# A Comprehensive Study of Gradient Conditions for Deep Proteome Discovery in a Complex Protein Matrix

**DOI:** 10.3390/ijms231911714

**Published:** 2022-10-03

**Authors:** Xing Wei, Pei N. Liu, Brian P. Mooney, Thao Thi Nguyen, C. Michael Greenlief

**Affiliations:** 1 Department of Chemistry, University of Missouri-Columbia, Columbia, MO 65211, USA; 2 Gehrke Proteomics Center, Christopher S. Bond Life Sciences Center, University of Missouri-Columbia, Columbia, MO 65211, USA; 3 Division of Biochemistry, University of Missouri-Columbia, Columbia, MO 65211, USA

**Keywords:** mass spectrometry, bottom–up proteomics, liquid chromatography gradient, HeLa cells

## Abstract

Bottom–up mass-spectrometry-based proteomics is a well-developed technology based on complex peptide mixtures from proteolytic cleavage of proteins and is widely applied in protein identification, characterization, and quantitation. A tims-ToF mass spectrometer is an excellent platform for bottom–up proteomics studies due to its rapid acquisition with high sensitivity. It remains challenging for bottom–up proteomics approaches to achieve 100% proteome coverage. Liquid chromatography (LC) is commonly used prior to mass spectrometry (MS) analysis to fractionate peptide mixtures, and the LC gradient can affect the peptide fractionation and proteome coverage. We investigated the effects of gradient type and time duration to find optimal gradient conditions. Five gradient types (linear, logarithm-like, exponent-like, stepwise, and step-linear), three different gradient lengths (22 min, 44 min, and 66 min), two sample loading amounts (100 ng and 200 ng), and two loading conditions (the use of trap column and no trap column) were studied. The effect of these chromatography variables on protein groups, peptides, and spectral counts using HeLa cell digests was explored. The results indicate that (1) a step-linear gradient performs best among the five gradient types studied; (2) the optimal gradient duration depends on protein sample loading amount; (3) the use of a trap column helps to enhance protein identification, especially low-abundance proteins; (4) MSFragger and PEAKS Studio have high similarity in protein group identification; (5) MSFragger identified more protein groups among the different gradient conditions compared to PEAKS Studio; and (6) combining results from both database search engines can expand identified protein groups by 9–11%.

## 1. Introduction

The term proteomics derives from the concept of proteome, which is a combination of protein and genome. Proteomics is a discipline to systematically study the sequence, structure, quantities, location, post translational modification (PTM), interaction, and functions of all proteins generated by a cell, tissue or biofluid under a specific condition [1,2]. Proteomics plays a significant role in drug discovery, biomarker research, and cancer screening [3]. Proteomic technologies are comprised of three critical parts: protein and peptide fractionation, mass spectrometry analysis, and bioinformatic database search and interpretation [4]. Protein and peptide fractionation techniques mainly include electrophoresis (i.e., isoelectric focusing [IEF], 2D-PAGE) and chromatography (reverse-phase liquid chromatography [RPLC], size exclusion chromatography [SEC], and strong cation exchange chromatography [SCX]) [5,6]. A variety of hybrid mass analyzers are commonly used for protein and peptide analysis [7]. Proteome database searches are performed based on either de novo sequencing or precursor peptide mass, and fragment mass information with protein databases or spectral libraries using search engines such as MSFragger, PEAKS Studio, and Spectronaut.

Ion mobility spectrometry (IMS) is a technique separating gas-phase ions based on their collision cross section (CCS). IMS is capable of coupling to mass spectrometry (MS) due to its small size, high efficiency, low voltage requirement, and compatibility with other separation methods [8]. Coupling IMS to MS grants an additional separation dimension to improve separation efficiency and promote deeper proteome coverage.8 Commercial IMS-MS systems include FAIMS (Owlstone and Thermo), TWIMS-MS (Waters), DT-IMS-MS (Agilent, Tofwerk, and Excellims), PF-IMS-MS (IonWorks), DMS (Sionex and AB Sciex), and TIMS-MS (Bruker Daltonics) [9].

Among these IMS systems, the tims-ToF pro (Bruker) stands out in deepening the proteome coverage due to its dual trapped IMS (tims), parallel accumulation serial fragmentation (PASEF) scan mode, and synchronized quadruple [8,10]. The tims PASEF scan mode enables accumulation of peptide ions in first section, perform CCS-based separation, and serial elution of separated ions in the second section, which achieves zero ion loss and maintains high sensitivity. The synchronized quadruple enhances sequencing speed. Therefore, the tims-ToF system features fast scanning speeds without sacrificing sensitivity, and functions as a promising technique for deeper proteome coverage and label free quantitation [10,11].

Nano-LC fractionation is usually coupled to IMS-MS, functioning as the first-stage online separation of peptides prior to further ion mobility- and mass-based separations. A trap column is commonly used as a precolumn prior to nano-LC for large sample volume analysis, as well as fast loading and desalting [12,13]. The use of a gradient is a key factor affecting LC separation performance as well as improving proteome coverage. Retention time prediction models can help predict retention times and facilitate the building of appropriate liquid chromatographic gradients for screening therapeutic peptides and targeted proteomics studies [14,15,16]. However, this model requires the known structure of analytes (e.g., amino acid composition, peptide sequence, and physicochemical properties) [17], which makes it challenging to apply in untargeted proteomics studies as untargeted proteomics studies the global proteins and does not target a specific protein or peptides. Untargeted proteomics is also called discovery proteomics, and deep proteome profiling is one of challenges it faces [5]. Common nano-LC gradients used in discovery proteomics are single-stage linear or multi-stage linear [18,19,20,21]. It is worthwhile to explore various gradient conditions to optimize the proteome coverage for tims-ToF-based proteomics studies. Here, we investigate the effect of five common gradient types (linear, logarithm-like, exponent-like, stepwise, and step-linear), three gradient time courses (22 min, 44 min, and 66 min), two sample loading amounts (100 ng and 200 ng), and two separation conditions (use of a trap column and no trap column) to determine their influence on protein group and unique peptide identification in HeLa cell digests.

PEAKS Studio [22] operates based on both a de novo sequencing algorithm and database search algorithm. De novo sequencing enables discovery of potential novel peptides. Its decoy-fusion strategy ensures an accurate and sensitive database search. MSFragger is a fast, sensitive, and comprehensive database search engine. As its database search is based on peptide ion index and fragment ion index, it significantly reduces the total analysis time for the peptide search and uses a similarity calculation between experimental and theoretical spectra candidates [23,24,25]. The PEAKS Studio and MSFragger search engines were compared at the level of protein group and unique peptide identification under consensus search parameters.

## 2. Results and Discussion

### 2.1. Gradient Types and Search Engines

**Figure 1:**
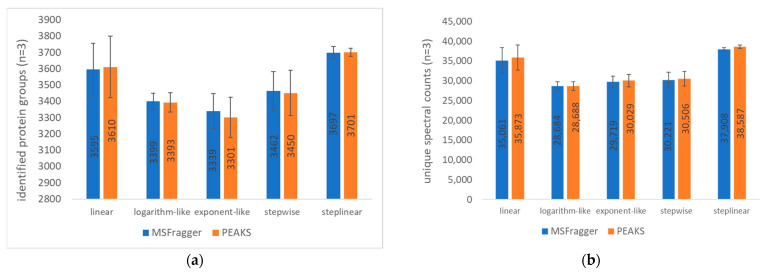
Comparison of the 44 min gradient types and search engines. MSFragger and PEAKS Studio were used to search data from a 100 ng HeLa digest on-column loading. (**a**) Number of identified protein groups; (**b**) number of unique spectral counts. The mean and standard deviation of triplicate injections are shown for each of 5 gradient types by search engines.

When we look at the MSFragger-based results (blue bars) among gradient types (Figure 1a,b; discussed and shown in Section 3.5), the step-linear gradient performed best with highest number (3697) of identified protein groups and 38,587 unique spectral counts; linear gradient ranked second with 3595 identified protein groups and 35,061 unique spectral counts; stepwise, logarithm-like and exponent-like gradient had <3500 protein groups and <30,250 unique spectral counts identified, of which the exponent-like gradient and logarithm-like gradient generated the least number of identified protein groups (3339) and unique spectral counts (28,684), respectively. Therefore, step-linear gradient works best for optimal protein group identification under our conditions.

PEAKS Studio gave similar results to the MSFragger search engine for the number of identified protein groups and unique spectral counts across all gradient conditions considering the approximate numbers of identified protein groups and unique spectral counts between the two search engines and their overlapping standard deviation bars, indicating the similarity in search power between the two search engines.

**Figure 2:**
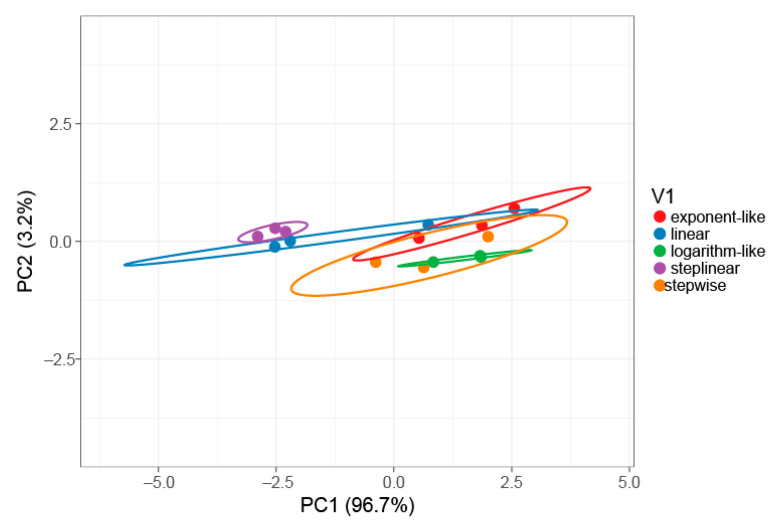
PCA plot of five different gradient types. Prediction ellipses represent the 95% confidence interval. PCA analysis was performed using the ClustVis tool [26]. The PCA was blinded to four sets of data, namely protein groups identified using MSFragger (MSFragger_PG), protein groups (PG) identified using PEAKS Studio (PEAKS_PG), unique spectral counts (USC) from MSFragger output (MSFragger_USC), unique spectral counts from PEAKS Studio (PEAKS_USC) (Table S1).

To verify the statistical difference in protein identification among five gradient types, principal component analysis (PCA) was performed (Figure 2). The principal components PC1 and PC2 explain 96.7% and 3.2% of the total variance, respectively. The results of the analysis show that a step-linear gradient (purple) is clearly separated from exponent-like (red), logarithm-like (green) and stepwise (orange) gradients along PC1. A distinguishable separation is observed between the stepwise gradient (purple) and linear gradient (blue) along PC2, indicating that the step-linear gradient type is statistically different from the other gradient types in for the number of identified proteins.

**Figure 3:**
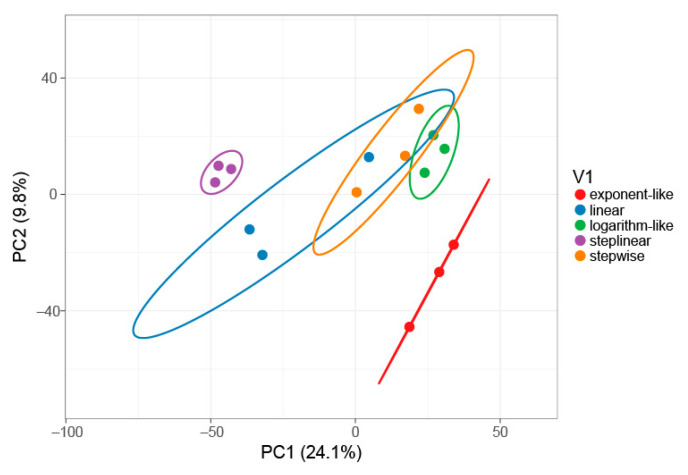
PCA plot of the five different gradient types based on all 4127 proteins identified across all five gradients. Prediction ellipses represent the 95% confidence interval. PCA analysis was performed using ClustVis tool [26]. The PCA was blinded to the unique spectral counts of all 4127 proteins identified across all five gradients using MSFragger (Table S2).

To investigate how the identified proteins differentiate between gradient types, a PCA plot based upon the unique spectral counts of 4127 proteins identified across all five gradients using MSFragger was made in Figure 3. The results also show that the step-linear gradient (purple) is clearly separated from the other four gradient types, indicating that the step-linear gradient type is statistically different from the other gradient types in protein identification.

**Figure 4:**
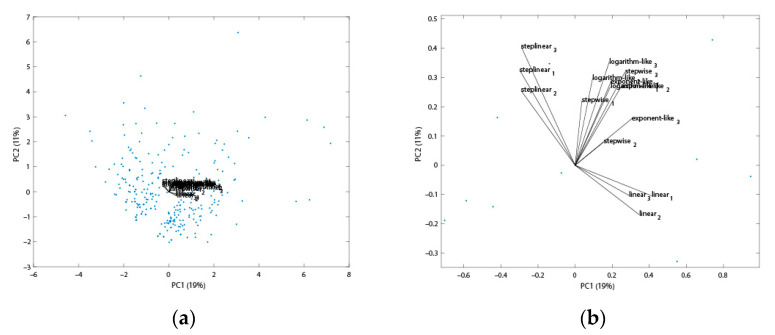
PCA biplot of the five different gradient types based on 274 proteins which were only either identified by the linear or step-linear gradient. (**a**) Zoom-out view, in which blue dots represent 274 proteins, the central part is the load plot; (**b**) zoom-in view of loading plot. The PCA was blinded to the 274 proteins which were either identified by the linear or step-linear gradient using MSFragger (Table S3).

Further, the proteins only identified by either a linear or step-linear gradient and how it may affect the PCA analysis were explored. A PCA biplot based upon 247 proteins (only identified either by the step-linear or linear gradient) is shown in Figure 4. The loading plot (Figure 4b) shows the angle between triplicate step-linear loads and linear loads are larger than 90°, suggesting that the two gradients are negatively correlated.

**Figure 5:**
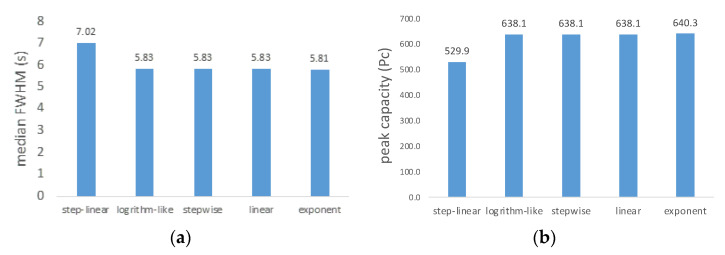
Comparison of (**a**) median FWHM and (**b**) peptide peak capacity among five gradient types. The median FWHM was calculated based on all peptides (with a minimum intensity of 2500) using MaxQuant (version 2.0.3.1). Peptide peak capacity was calculated by dividing the run time by the median FWHM. Ion chromatograms are shown in Figures S1–S12.

As shown in Figure 5, peak width and peak capacity were studied for the five gradient types. The results indicate the step-linear gradient has a full width at half maximum (FWHM) of 7.02 s and a peak capacity of 529.9, which are highest and lowest, respectively, among the five gradient types. Usually, peak capacity is related to protein identification, but the results obtained here did not show a strong relationship. In fact, we observed that the step-linear gradient has highest FWHM and protein identification, but lowest peak capacity. This may be due to its larger peak width resulting in improved sampling opportunity, and corresponding increased protein identification [16]. As shown in Figures S1–S6, the peptides elution profiles are different from one another. We observe that peptides in all profiles elute between 12% B and 80% B. The linear gradient chromatogram (Figure S1) has a significant number of peptides eluting between 30 and 44 min, while the step-linear gradient (Figure S2) shows peptides were more evenly eluted between 15 and 50 min. Compared with linear gradient (ramp from 3 to 37% over 0–44 min), the step-linear gradient has three sections of gradient. In the first section (ramp 3–17% over 0–14 min), the gradient is steeper than linear gradient, which leads to faster elution of peptides. In the second section, the gradient (ramp from 17 to 25% over 14–34 min) is shallower than linear gradient, giving a better separation of peptides at the expense of wider chromatographic peaks and lower intensity. This result explains why the step-linear gradient has wider median peak widths, but the highest number of protein groups identified. The final section of the gradient (ramp from 25 to 37% over 34–44 min) is shared with all gradient types and elution of the remaining peptides is similar.

**Figure 6:**
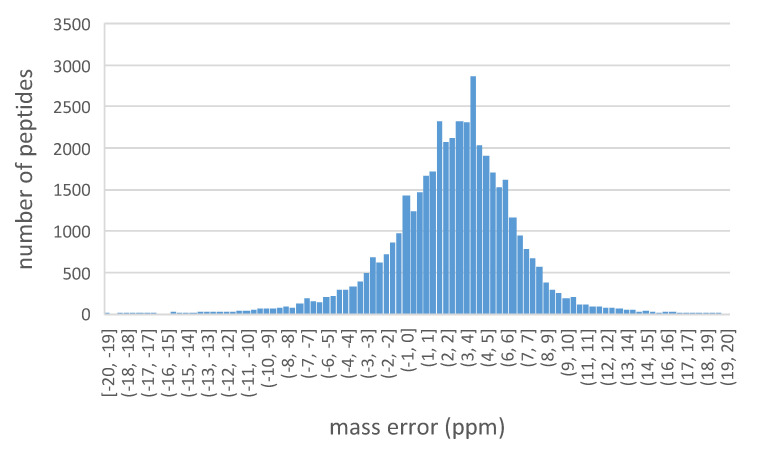
Mass errors of precursor peptides. Histograms illustrate the distribution of the number of peptides as reflected by precursor mass error (ppm) combined from all five gradient types.

The distribution of the precursor peptides mass error is shown in Figure 6. The histogram indicates the mass error of all peptides are within 20 ppm. Among those, the peptides with mass error more than −4 ppm, but no less than 10 ppm, account for 93.7% of all detected peptides. 

**Figure 7:**
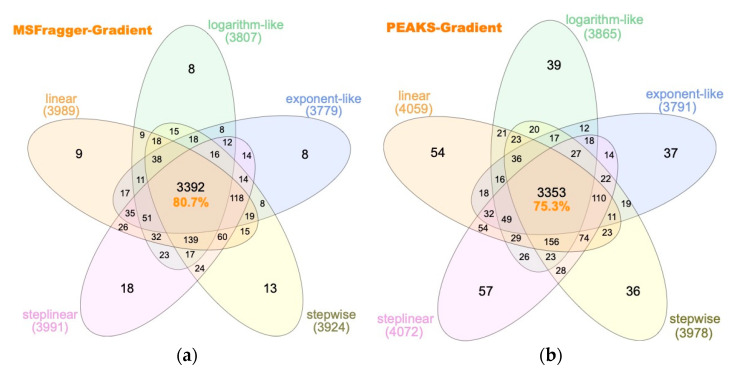
Comparison by gradient types and search engines using a 100 ng HeLa digest on-column loading. (**a**) Venn diagram showing identified protein groups using MSFragger, (**b**) Venn diagram showing identified protein groups using PEAKS Studio.

From the MSFragger-based overlapping protein groups (Figure 7a), the 5 gradient types have 3392 common protein groups, accounting for 80.7% of total identified across all gradient types (4205), demonstrating ~80% similarity between the 5 gradients in protein identification on the one hand, and ~20% potential contributions the selection of optimal gradient types may make in protein identification.

Compared to 80.7% common proteins from MSFragger, PEAKS Studio identifies 75.3% overlapping protein groups across different gradient types, 39 protein groups less than PEAKS Studio, which may be attributed to the difference in the results filtering algorithm between the two search engines [27].

Gradient types were sorted by the number of identified unique protein groups in the following descending order. For MSFragger, the order is step-linear (18) > stepwise (13) > linear (9) > exponent-like (8) = logarithm-like (8); for PEAKS Studio, the order is step-linear (57) > linear (54) > stepwise (39) > logarithm-like (37) > exponent-like (36). The step-linear gradient has the most unique protein groups identified in both search engines, suggesting that the step-linear gradient holds the most promises for deepening proteome coverage.

### 2.2. Gradient Durations, Loading Amount and Search Engines

#### 2.2.1. Gradient Durations and Loading Amount

**Figure 8:**
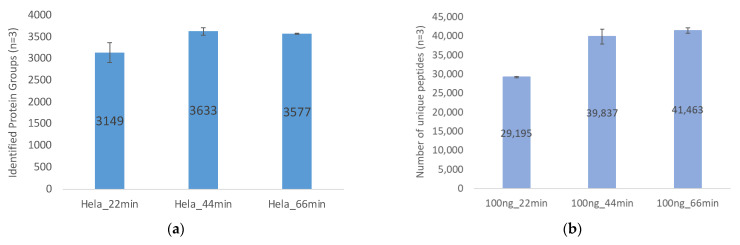

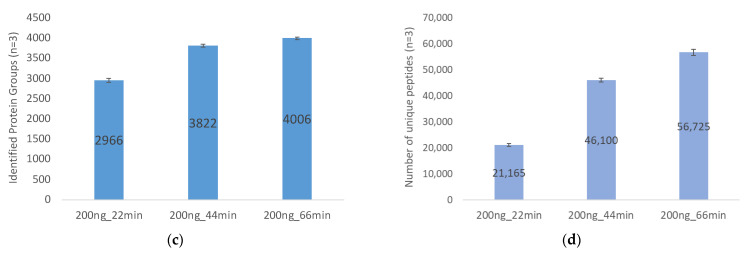
Proteome coverage compared to gradient. A comparison of gradient time course and HeLa digest loading amount is presented: (**a**) Identified protein groups using 100 ng of HeLa digest; (**b**) unique spectral counts using 100 ng of HeLa digest; (**c**) identified protein groups using 200 ng of HeLa digest; (**d**) unique spectral counts using 200 ng of HeLa digest. The mean and standard deviation of triplicate injections are shown for each of 3 gradient durations by search engines. (Note: 1. The search engine used was MSFragger; 2. In total, 200 ng datasets were collected using a different nanoLC column from that for 100 ng datasets, which explains why the 22 min gradient of 200 ng HeLa digest loading (Figure 8c,d) had lower number of protein groups and unique spectral counts identified than that of 100 ng of HeLa digest loading (Figure 8a,b)).

Optimal gradient durations were further studied based upon the step-linear gradient, which was indicated as the optimum gradient type for protein identification. As shown in Figure 8a,b, using 100 ng of HeLa digest, the 44 min gradient (62 min total run time, dataset searched with MSFragger) gave the best optimal performance. When the amount of HeLa digest was increased to 200 ng (Figure 8d), the 66 min gradient (90 min run time) yielded the best protein identification.

In Figure 8a,b for loading 100 ng of HeLa digest on column, the 22 min gradient identified 3149 protein groups and 29,195 unique spectral counts, respectively, which were 13% and 27% less than the 44 min gradient (3633 protein groups and 39,837 unique spectral counts). That said, 44 min and 66 min gradients had similar amounts of identified protein groups (~3600) and unique spectral counts (~40,000) with overlapping standard deviation bars. Thus, for 100 ng HeLa digest analysis, 44 min gradient is the optimal time course to maximize protein group identification covering the most unique spectral counts considering its performance and time efficiency. The possible reason why 44 min gradient identified similar amounts of proteins compared with 66 min gradient for 100 ng peptides loading is that 44 min gradient is enough to have the 100 ng peptide mixtures well separated, and extending the gradient length to 66 min would perform more work such as widening the peak width and lowering the intensity [16] (bringing some peaks below detectable threshold) rather than enacting better separation (have some new peaks show up). 

In Figure 8c, based upon 200 ng of HeLa digest, the 66 min gradient identified 4006 protein groups, 35% more compared to the 22 min gradient (2966 protein groups), and a 5% increase over the 44 min gradient (3822 protein groups). In Figure 8d, regarding unique spectral counts, unique spectral counts for the 66 min gradient were 56,725, and 168% higher than the 22 min gradient (21,165 unique spectral counts), and 23% more than the 44 min gradient (46,100 unique spectral counts). Therefore, for the 200 ng HeLa digest analysis, the 66 min gradient is optimal for protein identification. In summary, optimal gradient time depends on protein loading amount. The 44 min gradient yields optimal performance for 100 ng protein analysis, while the 66 min gradient works best for 200 ng protein analysis.

**Figure 9:**
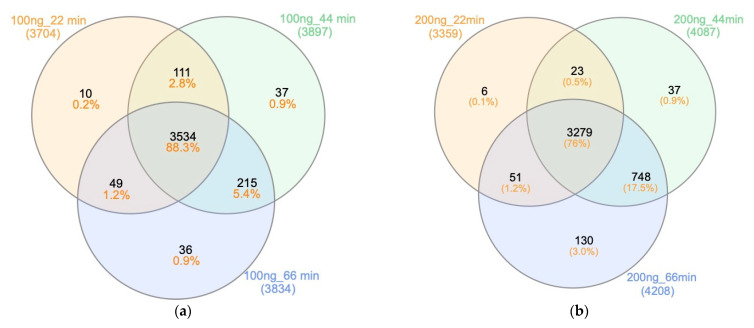
Proteome overlap between gradients. MSFragger-based Venn diagrams showing common identified protein groups between three gradient time courses and load amounts of HeLa digest (**a**) using 100 ng of HeLa digest; (**b**) using 200 ng of HeLa digest.

The Venn diagrams in Figure 9 summarize the MSFragger-based results of the different gradient times and sample loading. The 100 ng and 200 ng Hela digest loading, respectively, identified 88.3% and 76% overlapping protein groups across the different gradient durations. Despite the high degree of similarity, each gradient duration identified their distinct protein groups, which were not identified by other time durations. For example, in Figure 9a for 100 ng HeLa digest analysis, 10 unique protein groups in 22 min gradient were not identified in either 44 min or 66 min gradients. This is mainly attributed to the data-dependent acquisition (DDA) mode used for data acquisition, which only focuses on the most abundant peptide ions, resulting in a number of missing values and lower reproducibility [28].

Across all gradient durations, number of identified unique protein groups show the following trend for MSFragger:

Loading 100 ng of HeLa digest (Figure 8a), 44 min (37) > 66 min (36) > 22 min (10);

Loading 200 ng of HeLa digest (Figure 8c), 66 min (130) > 44 min (37) > 22 min (6).

In the 100 ng HeLa digest analysis, the 44 min gradient has the largest number of unique protein groups identified. When increasing the amount of HeLa digest to 200 ng, the 66 min gradient performs best, providing further evidence that optimal gradient for deepening proteome coverage is related to the amount of sample loaded to the LC/MSMS.

#### 2.2.2. Search Engines

**Figure 10:**
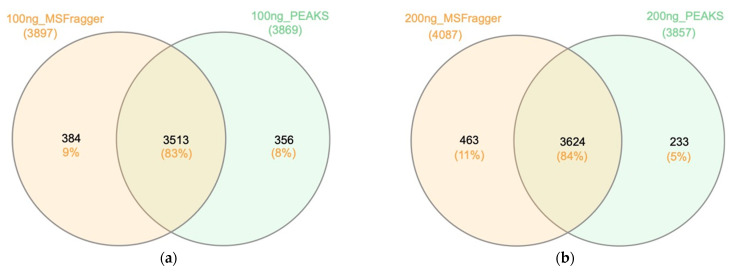
Proteome overlap of two search engines. Venn diagram showing overlapping identified protein groups between MSFragger and PEAKS Studio for the data acquired under a 44 min step-linear gradient (**a**) using 100 ng of HeLa digest; and (**b**) using 200 ng of HeLa digest.

In Figure 10a, 100 ng of HeLa digest was analyzed under a 44 min step-linear gradient (62 min run time). MSFragger and PEAKS Studio were used to examine the results and have 83% (3513) overlapping protein groups, indicating the high similarity of the two search engines. The unique protein groups identified by MSFragger and PEAKS Studio were 9% and 8%, respectively. MSFragger identified slightly higher numbers of unique protein groups compared to PEAKS Studio, and the combination of both database search engines could expand the identified protein groups by up to 9%. (The 200 ng protein analysis in Figure 10b shows consistent results with Figure 10a).

### 2.3. The Use of Trap Column Versus No-Trap Column

**Figure 11:**
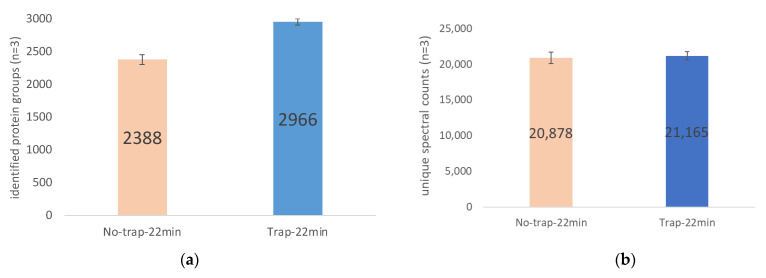
Trap column and proteome coverage. Comparisons of proteome coverage were made with and without a trap column loaded with 200 ng of HeLa digest (**a**) identified protein groups; (**b**) unique spectral counts. The mean and standard deviation of triplicate injections are shown for the inclusion and exclusion of a trap column.

The effect of using a trap column on protein identification was studied as well. The results in Figure 11 show that coupling a trap column prior to the analytical column improves protein identification. As shown in Figure 11a,b for 22 min gradient, the incorporation of the trap column increased the number of identified protein groups by 24.2% from 2388 to 2966; the number of unique spectral counts increased 1.4% from 20,878 to 21,165.

**Figure 12:**
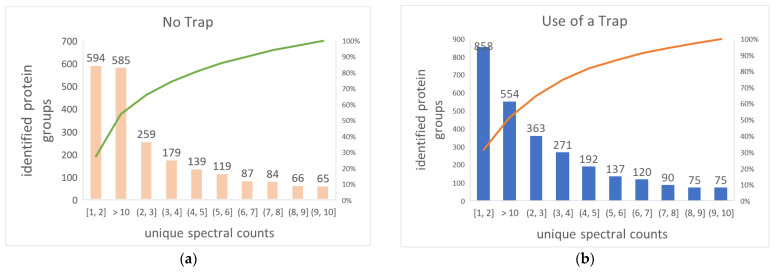
Low-abundance proteins and unique spectral counts. Histograms illustrate the distribution of identified proteins as reflected by respective covered unique spectral counts (**a**) no trap column; (**b**) inclusion of a trap column on the column. The mean and standard deviation of triplicate injections are shown for the number of identified protein groups covering varying number of unique spectral counts.

Figure 12a,b elucidate the distribution of identified proteins by the number of unique spectral counts. When a trap column was used on the analytical column, the number of identified proteins covering 1–2 unique spectral counts increased by 336, accounting for 58% of total identified protein increasement; identified proteins covering 2–10 unique spectra were up by 266, making up 46% of total increased identified protein; the number of proteins with >10 unique spectral counts decreased by 24, which equaled 4% of the total increased number of identified proteins. In summary, the inclusion of a trap column on analytical column yields more proteins, with ≤10 unique spectral counts identified, but sees a drop in identified proteins with >10 unique spectral counts. This explains why only a 1.4% increase in unique spectral counts contributed to 24.2% increase in the number of identified proteins. This is most likely due to the trap column’s focusing effect on peptides, 24 which narrow peak width, raising the signal of low-abundance proteins above the detectable threshold while reducing the total number of scans across the peaks of high-abundance proteins due to fixed time per scan but narrowed peak width.

## 3. Materials and Methods

### 3.1. HeLa Digest

Pierce™ HeLa Protein Digest Standard (Catalog number: 88328) were reconstituted to 100 ng/μL in 18.2 MΩ·cm water with 5% acetonitrile (ACN) and 0.1% formic acid for nano-LC-tims-ToF Pro analysis.

### 3.2. Nano-LC

A Bruker nanoElute system (self-packed and pulled needle analytical column, 20 cm × 75 μm id, Waters BEH C18, 1.7 μm) was coupled between a trap column (Thermo trap cartridge, 5 mm × 0.3 mm id, C18, 5 μm) and a dual trapped ion mobility spectrometer coupled to a quadruple-time of flight mass spectrometer system (Bruker tims-ToF Pro, Bremen, Germany). Binary mobile phases (A: 18.2 MΩ·cm water with 0.1% formic acid; B: ACN with 0.1% formic acid) were employed at the flow rate of 300 nL/min. Column and sample temperature were set at 50 °C and 4 °C, respectively. The injection volume was 1 μL.

### 3.3. Nano Electrospray Ionization (NSI)

A Bruker CaptiveSpray source is interfaced between the nanoLC and tims-ToF. It was operated in the positive ion mode. The capillary voltage was 1.8 kV and the end plate offset was 500 V. The nebulizer gas pressure was 0.4 bar. The dry gas flow rate was 3 L/min, and the dry temperature was 180 °C.

### 3.4. Tims-Q-ToF

The tims was performed under the parallel accumulation serial fragmentation (PASEF) mode. The inverse ion mobility (1/K_0_) ranged from 0.6 to 1.6 V·s/cm^2^. The Q-ToF mass-to-charge ratio (*m*/*z*) range was set from 100 to 1700. PASEF and tims were set to “on”. One scanning cycle includes one 100 ms MS and ten 100 ms PASEF frames. Each 100 ms PASEF frame acquires ten MS/MS spectra. A rolling collision energy from 76 to 123% of 42.0 eV was employed. An active exclusion method was applied with a release after 0.4 min. If the intensity ratio of a given precursor (within mass width error of 0.015 Da) to a previous precursor is more than 4, the precursor will be acquired for a second MS/MS spectrum. For *m*/*z* below 700 Da, the isolation width was set to 2 Da. For mass ranges from 800 to 1500 Da, the isolation width increased to 3 Da. Both the tims elution voltage and Q-ToF were calibrated using Agilent ESI-L Tuning Mix (Catalog number: G1969-85000).

### 3.5. Liquid Chromatography Gradients

**Figure 13:**
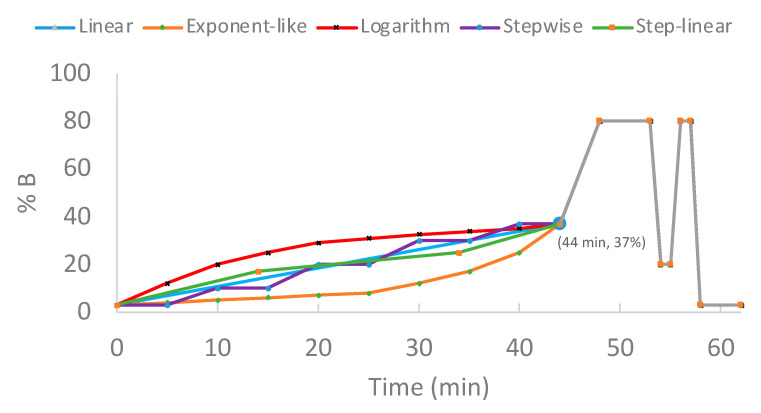
Gradient structures. A set of 44 min gradient types (62 min run time) were comprised of linear (blue), exponent-like (orange), logarithm-like (red), stepwise (purple), step-linear (green). For the first 44 min, linear had a gradient of 3–37% B over 44 min; exponent-like had a gradient of 3–8% B over 25 min, three 5 min serial gradients to 12% B, 17% B, and 25% B, prior to 37% B for 4 min; logarithm-like had eight 5 min serial gradients from 3% B, to 12% B, 20% B, 25%, 29% B, 31% B, 32.5% B, 33.9% B, and 35% B, prior to 37% B for 4 min; stepwise hold at initial 3% B for 5 min, gradient (5 min) to and hold (5 min) at 10%, 20%, 30%, and 37%, successively; step-linear had a gradient of 3–17% B over 14 min, 17–25% over 20 min, 25–37% over 10 min.

Five gradient profiles (linear, exponent-like, logarithm-like, stepwise, and step-linear) were investigated for protein groups and unique spectral counts. These experiments were conducted with trap column using 100 ng of HeLa digest on column. For all gradient profiles, the initial composition was 3% B (A: 18.2 MΩ·cm water with 0.1% formic acid; B: ACN with 0.1% formic acid), followed by 44 min ramps to 37% B in either linear, exponent-like, logarithm-like, stepwise, or step-linear (Figure 13). The gradient was then increased to 80% B over 4 min and held at 80% B for 5 min. Finally, a 7 min oscillating column wash was performed (1 min to 20% B/80% B/20% B, hold at 80% B for 1 min before ramping back to 3% B over 3 min). The oscillating section at the end of each gradient profile was designed and tested as an effective and quick approach to clean the analytical column. Compared to the end section of traditional gradient profile without an oscillating wash (e.g., stabilizing at 80% B isocratic for 9 min and then directly ramping to 3%), the oscillating wash in Figure 13 can be considered to have two additional minutes of gradient washes. As gradient elution can shorten the overall analysis time [29] (namely, carryover components elute out faster), the oscillation wash cleans column more effectively. Technical triplicates were acquired under each gradient profile.

**Figure 14:**
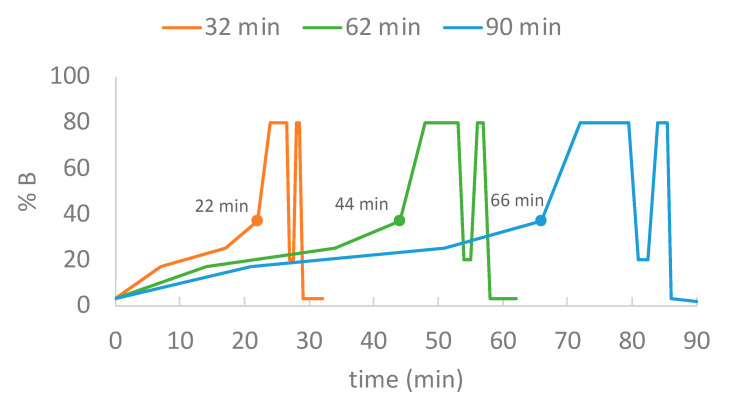
Step-linear gradient duration. The structures of the three step-linear gradients are shown for 22 min (orange 32 min run time), 44 min (green 62 min run time), and 66 min (blue 90 min run time).

Three-step-linear gradient durations (22 min, 44 min, and 66 min) were investigated for protein groups and unique spectral counts under two sets of on-column sample loading amounts (100 ng and 200 ng HeLa digest). These experiments were conducted with the inclusion of a trap column. As shown in Figure 14, for the 22 min gradient (orange), the initial composition was 3% B, followed by serial ramps to 17% B (7 min), 25% B (10 min), 37% B (5 min), prior to ramping (2 min) to and holding (2.5 min) at 80% B, followed by a 3.5 min oscillating column wash (0.5 min to 20% B/hold at 20% B for 0.5 min/0.5 min to 80% B/hold at 80% B for 0.5 min before ramping back to 3% B over 1.5 min). The 44 min and 66 min gradients were designed to double and triple the time of each section in 22 min gradient. To evaluate the impact of using trap column on protein identification, one additional 30 min gradient was added without trap column using 200 ng of HeLa digest. Technical triplicates were acquired under each condition.

### 3.6. Data Analysis

Raw data were searched against the Uniprot human database (20,577 entries, updated 3 August 2021) using both PEAKS Studio (PEAKS XPro) [22] and MSFragger (version 17.0) [23] search engines with consensus search parameters. An automated decoy database search was utilized to calculate discovery rate. Mass tolerances on precursor and fragment were ≤20 ppm and ≤0.1 Da, respectively; protein cleavage was set at “semispecific” with a maximum of 2 missed cleavages per peptide. Data type was DDA, and the enzyme was trypsin, cleaving after residue K and R but not before P. For post translational modification (PTM), carbamidomethylation (C) was the fixed PTM and oxidation (M) and acetylation (protein N-terminal) were set as variable PTM.

Exported datasets were further processed by removing duplicates based on the “protein group” (a group of proteins identified by the same set of peptides, and it cannot be determined these peptides are attributed to which of these proteins [30].) column and filtering proteins by a 1% protein FDR cutoff for the number of unique spectral counts under each condition. The number of identified protein groups were filtered by ≥1 unique spectral count per protein. Bar charts and Venn diagrams were plotted to compare the identified protein groups and unique spectral counts as well as the protein groups overlapping under different conditions. For the bar graphs, the number of protein groups identified were filtered based on the unique spectral counts of each technical replicate; while, for the Venn diagrams, the filtration was based upon the combined unique spectral counts across triplicate measurements.

## 4. Conclusions

This study refined the gradient conditions including gradient type, gradient duration, loading amount, and inclusion/exclusion of trap column to obtain optimal proteome coverage. Additionally, two database search engines (PEAKS Studio and MSFragger) were used and compared. The results indicate that step-linear gradient performed best among all tested gradients. Gradient durations depended on the loading amount, namely a 44 min gradient (62 min run time) worked best for 100 ng HeLa digest analysis (>3600 protein groups identified), while a 66 min gradient (90 min run time) was optimal for a 200 ng peptide analysis (>4000 protein groups identified). The use of a trap column improved protein identification, especially for low-abundance proteins. High similarities were observed between PEAKS Studio and MSFragger in protein identification and MSFrager featured higher numbers of overlapping protein groups compared to PEAKS Studio. The combination of both search engines was able to increase the total number of identified proteins by 9–11%. For this study, we focused gradient conditions while keeping the rest of the system constant (MS settings, chromatographic material, solvents, etc.). By keeping all the other conditions constant, the results show that the proteome coverage can be improved with changes to the gradient. 

## Data Availability

Original data are available upon request to the corresponding author.

## Supplementary Materials

The supporting information can be downloaded at: https://www.mdpi.com/article/10.3390/ijms231911714/s1.
