# Supplementary material for: A Comprehensive Study of Gradient Conditions for Deep Proteome Discovery in a Complex Protein Matrix
Source: Int J Mol Sci. 2022 Oct 3;23(19):11714. doi: 10.3390/ijms231911714 (PMC9569591; doi:10.3390/ijms231911714)
Supplement: Supplementary file 1 [file ijms-23-11714-s001.zip › Suppplementary information_Greenlief.pdf]

A Comprehensive Study of Gradient Conditions for Deep Proteome Discovery in a Complex Protein Matrix

Xing Wei [1,2], Pei N. Liu [2], Brian P. Mooney [2,3], Thao Thi Nguyen [2] and C. Michael Greenlief [1,2,*]

[1] Department of Chemistry, University of Missouri-Columbia, Columbia, Missouri 65211; xw4xf@mail.missouri.edu (X.W.)

[2] Gehrke Proteomics Center, Christopher S. Bond Life Sciences Center, University of Missouri-Columbia, Columbia, Missouri 65211; peiliu0824@gmail.com (P.L.), tnqp6@missouri.edu (T.T.N.)

[3] Division of Biochemistry, University of Missouri-Columbia, Columbia, MO 65211; mooneyb@missouri.edu

[*] Correspondence: greenliefm@missouri.edu; Tel.: +01-573-882-3288

Contents

Table S1: Data used for PCA plot

Figure S1: Base peak chromatogram and total ion current chromatogram of linear gradient type run 1 (top two), run 2 (middle two), and run 3 (bottom two).

Figure S2: Base peak chromatogram and total ion current chromatogram of step-linear gradient type run 1 (top two), run 2 (middle two), and run 3 (bottom two).

Figure S3: Base peak chromatogram and total ion current chromatogram of stepwise gradient type run 1 (top two), run 2 (middle two), and run 3 (bottom two).

Figure S4: Base peak chromatogram and total ion current chromatogram of logarithm-like gradient type run 1 (top two), run 2 (middle two), and run 3 (bottom two).

Figure S5: Base peak chromatogram and total ion current chromatogram of exponent-like gradient type run 1 (top two), run 2 (middle two), and run 3 (bottom two).

Figure S6: Base peak chromatogram and total ion current chromatogram of 22 min gradient (32 min run time) run 1 (top two), run 2 (middle two), and run 3 (bottom two) with 100 ng of HeLa digest loading.

Figure S7: Base peak chromatogram and total ion current chromatogram of 44 min gradient (62 min run time) run 1 (top two), run 2 (middle two), and run 3 (bottom two) with 100 ng of HeLa digest loading.

Figure S8: Base peak chromatogram and total ion current chromatogram of 66 min gradient (90 min run time) run 1 (top two), run 2 (middle two), and run 3 (bottom two) with 100 ng of HeLa digest loading.

Figure S9: Base peak chromatogram and total ion current chromatogram of 22 min gradient (32 min run time) run 1 (top two), run 2 (middle two), and run 3 (bottom two) with 200 ng of HeLa digest loading without a trap column.

Figure S10: Base peak chromatogram and total ion current chromatogram of 22 min gradient (32 min run time) run 1 (top two), run 2 (middle two), and run 3 (bottom two) with 200 ng of HeLa digest loading with a trap column.

Figure S11: Base peak chromatogram and total ion current chromatogram of 44 min gradient (62 min run time) run 1 (top two), run 2 (middle two), and run 3 (bottom two) with 200 ng of HeLa digest loading with a trap column.

Figure S12: Base peak chromatogram and total ion current chromatogram of 66 min gradient (90 min run time) run 1 (top two), run 2 (middle two), and run 3 (bottom two) with 200 ng of HeLa digest loading with a trap column.

Table S1. Data used for PCA plot

	linear_1	linear_2	linear_3	logarithm-like_1	logarithm-like_2	logarithm-like_3	exponent-like_1	exponent-like_2	exponent-like_3	stepwise_1	stepwise_2	stepwise_3	steplinear_1	steplinear_2	steplinear_3
	linear	linear	linear	logarithm-like	logarithm-like	logarithm-like	exponent-like	exponent-like	exponent-like	stepwise	stepwise	stepwise	steplinear	steplinear	steplinear
MSFragger_PG	3707	3667	3411	3459	3370	3369	3243	3321	3454	3335	3480	3572	3671	3679	3742
PEAKS_PG	3738	3700	3393	3462	3357	3359	3185	3287	3432	3293	3497	3560	3679	3696	3728
MSFragger_USC	37216	36685	31281	29866	28072	28114	28437	29378	31342	28448	29875	32341	37317	38046	38362
PEAKS_USC	37836	37555	32229	29936	28181	27946	28929	29372	31787	28739	30318	32460	38112	38610	39040

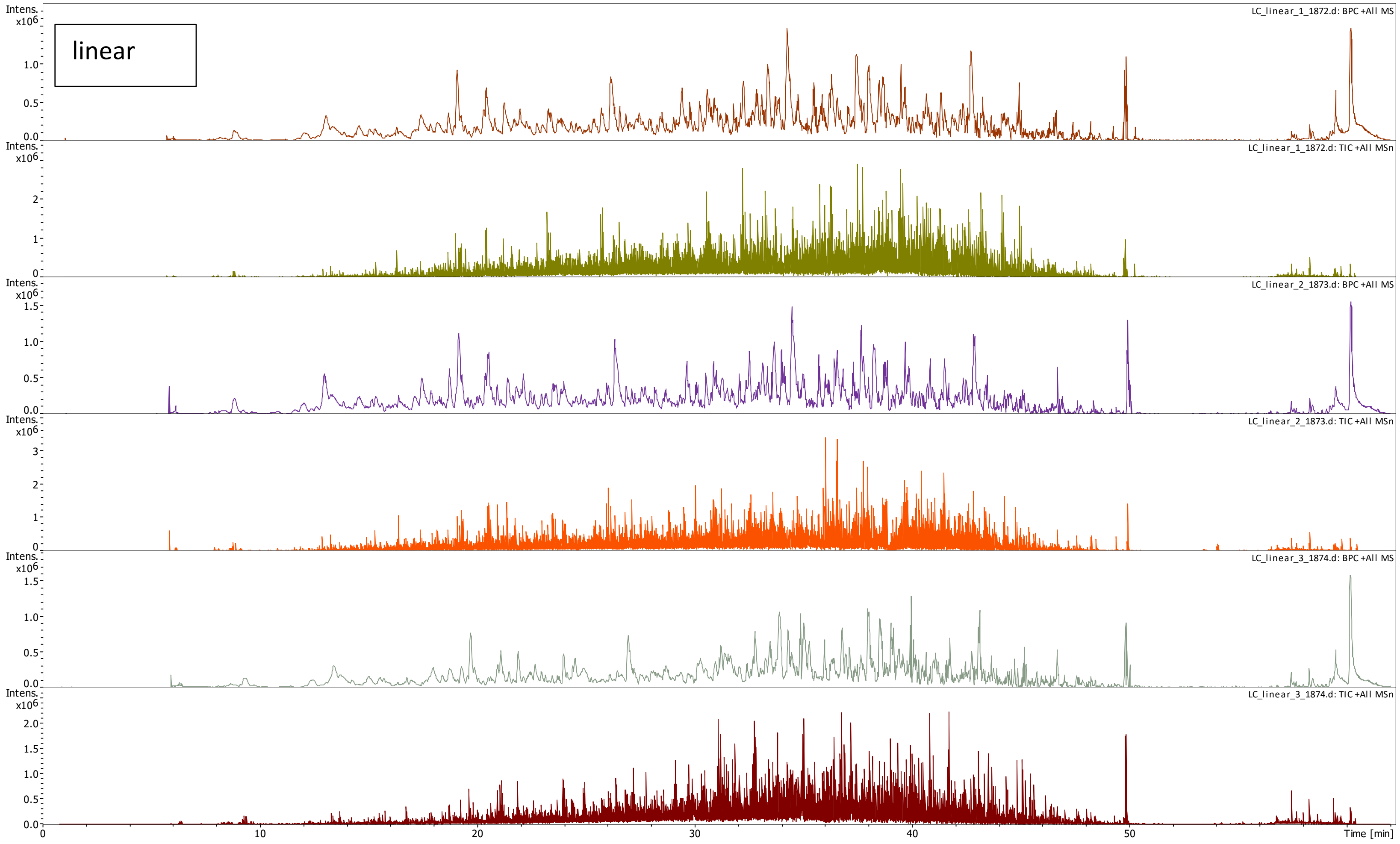

Figure S1. Base peak chromatogram and total ion current chromatogram of linear gradient type run 1 (top two), run 2 (middle two), and run 3 (bottom two).

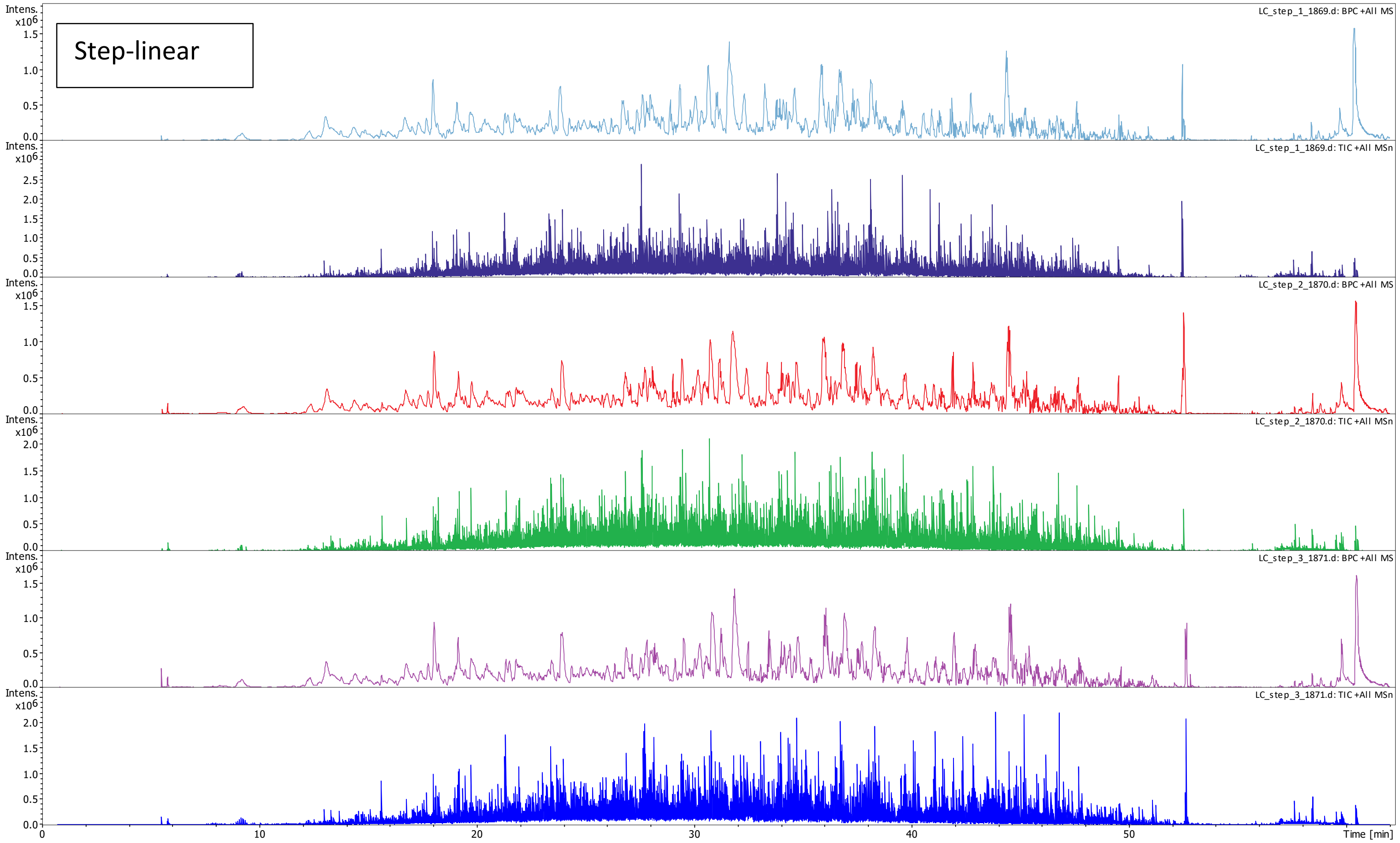

Figure S2. Base peak chromatogram and total ion current chromatogram of step-linear gradient type run 1 (top two), run 2 (middle two), and run 3 (bottom two).

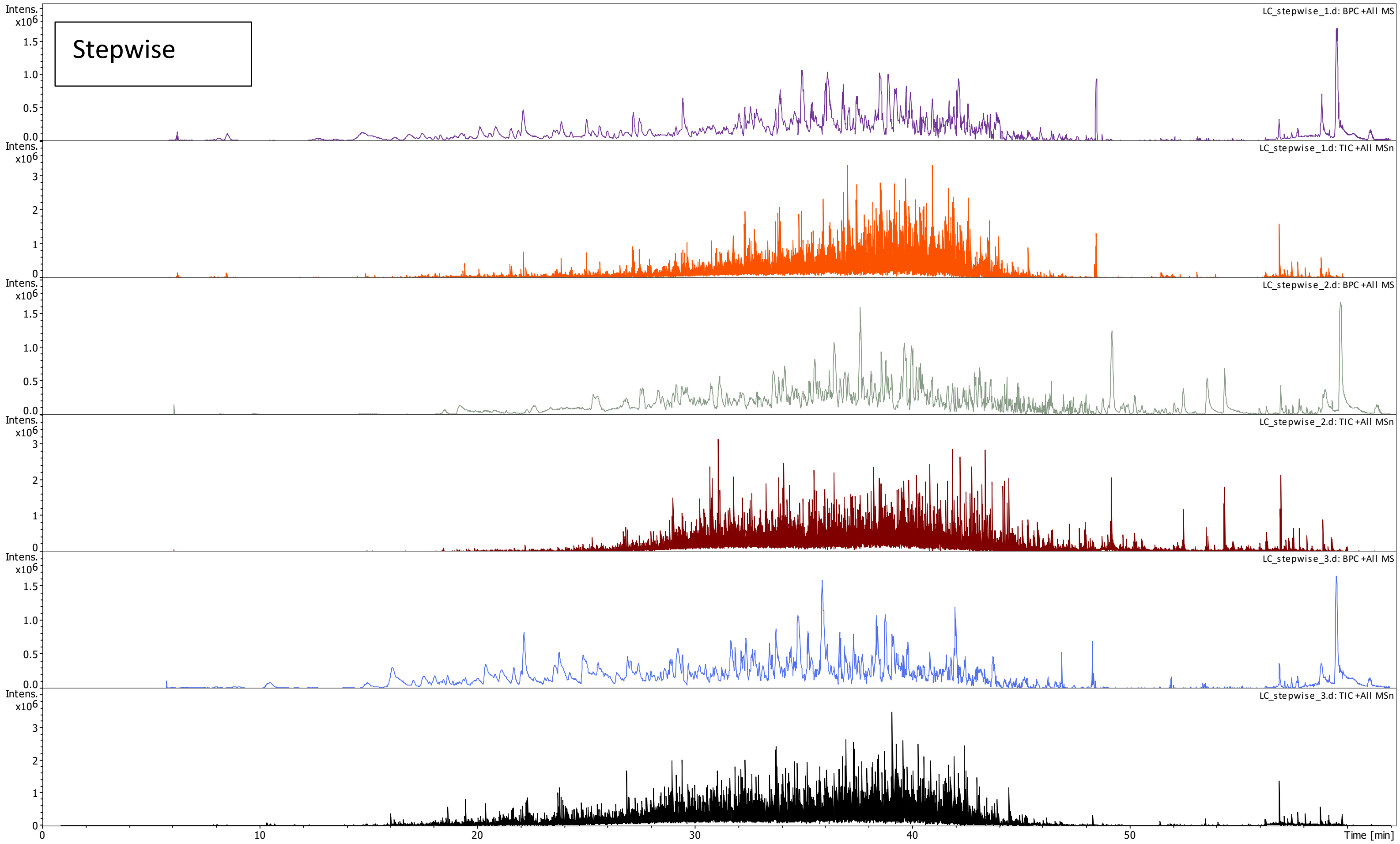

Figure S3. Base peak chromatogram and total ion current chromatogram of stepwise gradient type run 1 (top two), run 2 (middle two), and run 3 (bottom two).

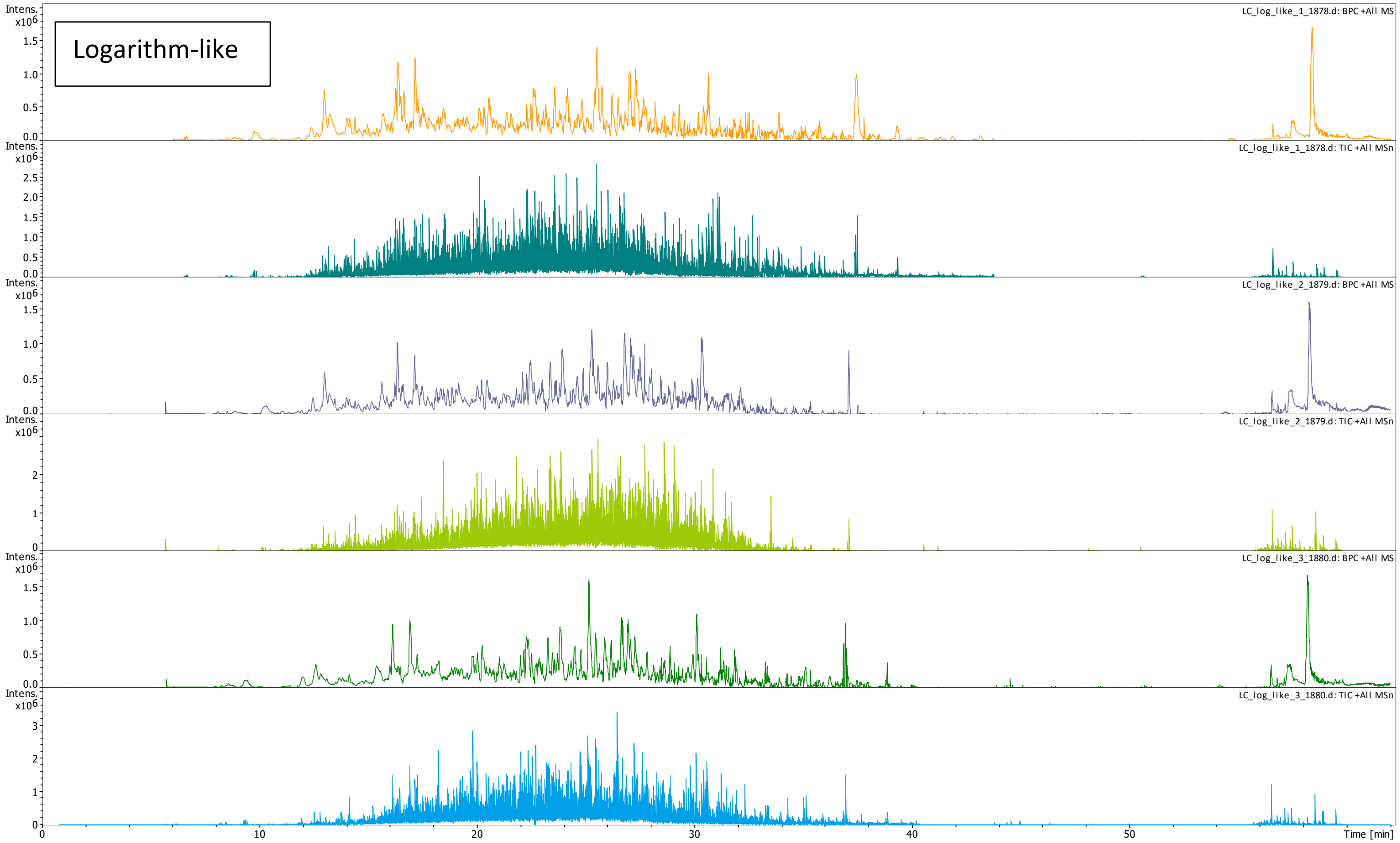

Figure S4. Base peak chromatogram and total ion current chromatogram of logarithm-like gradient type run 1 (top two), run 2 (middle two), and run 3 (bottom two).

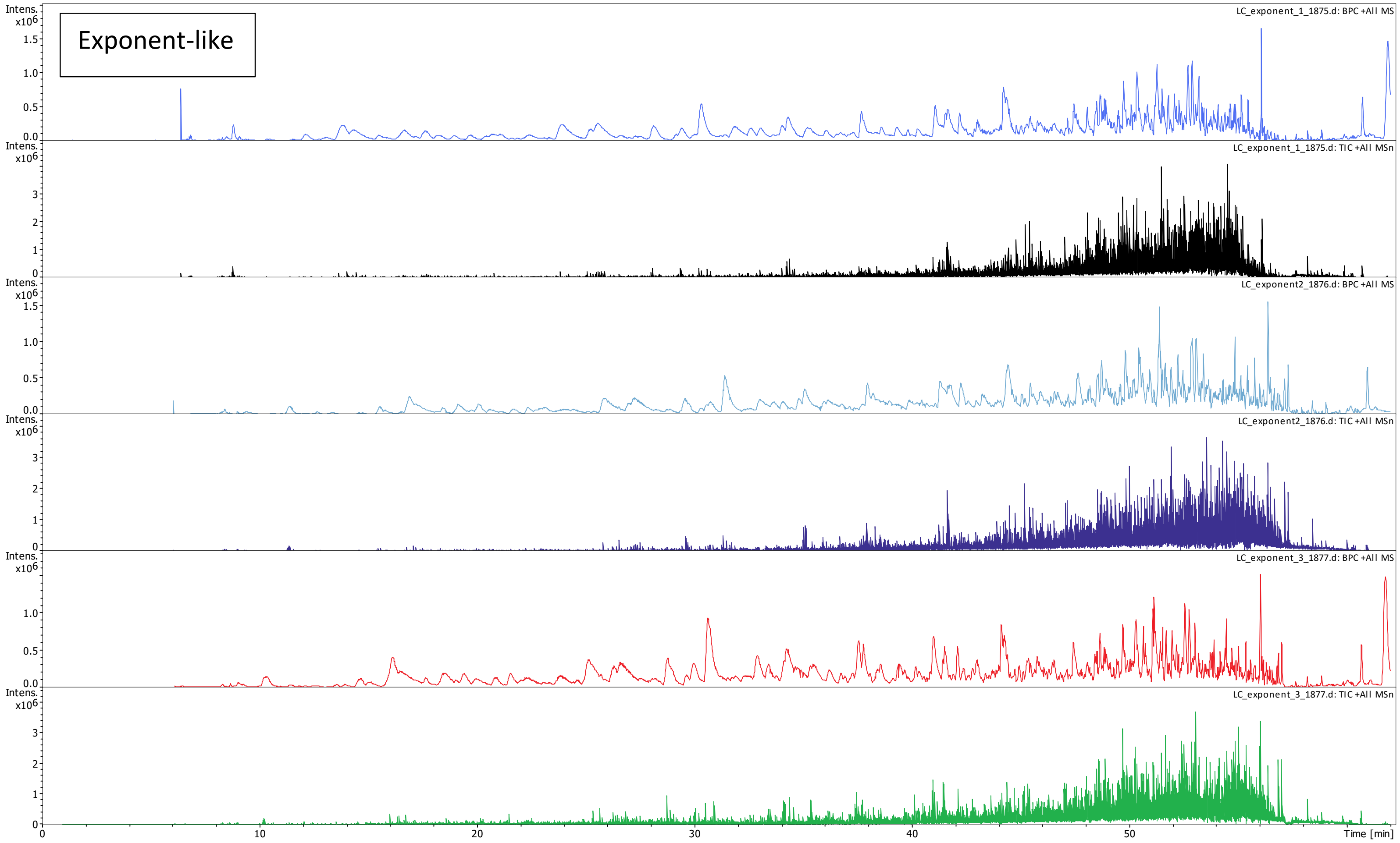

Figure S5. Base peak chromatogram and total ion current chromatogram of exponent-like gradient type run 1 (top two), run 2 (middle two), and run 3 (bottom two).

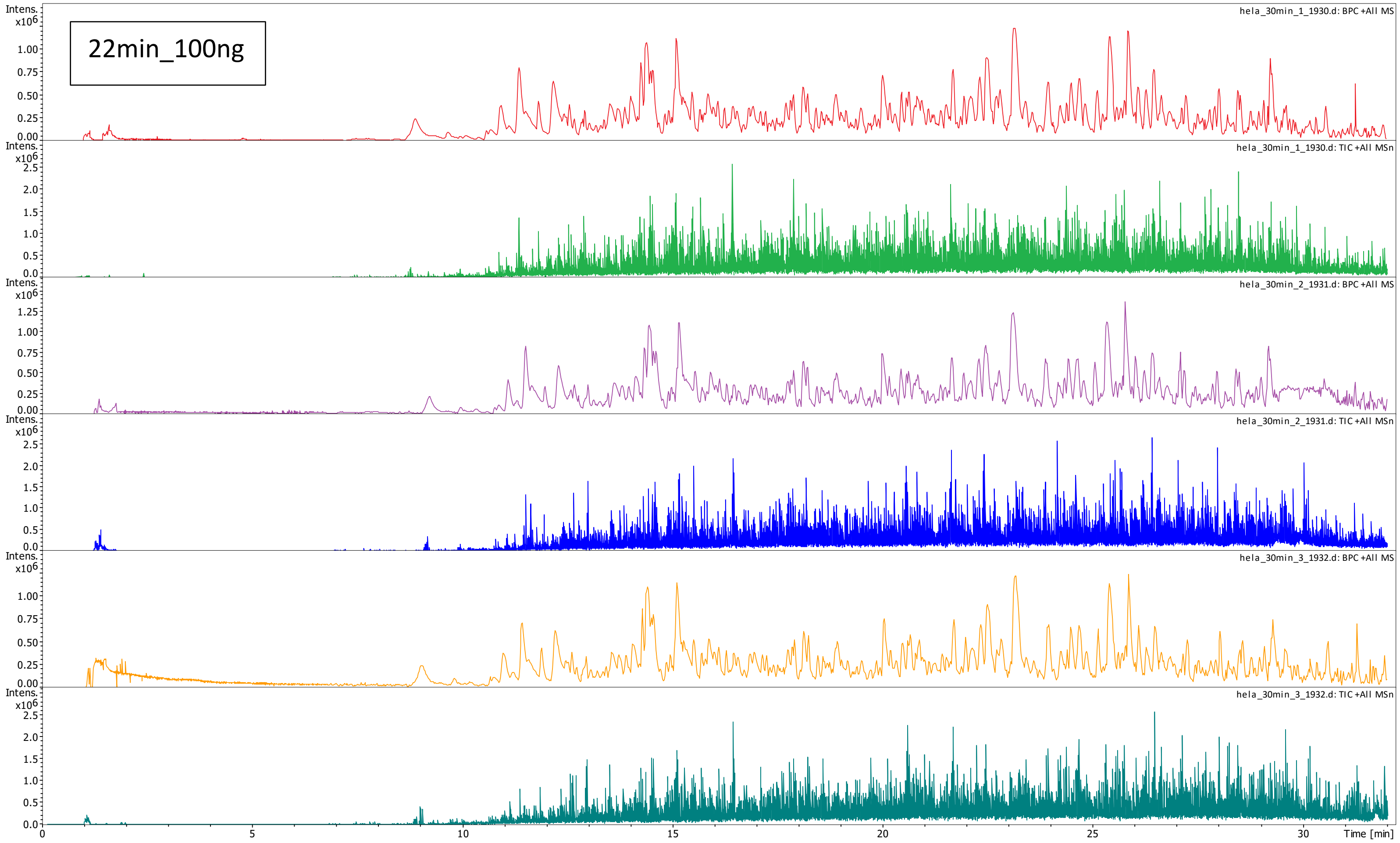

Figure S6. Base peak chromatogram and total ion current chromatogram of 22 min gradient (32 min run time) run 1 (top two), run 2 (middle two), and run 3 (bottom two) with 100 ng of HeLa digest loading.

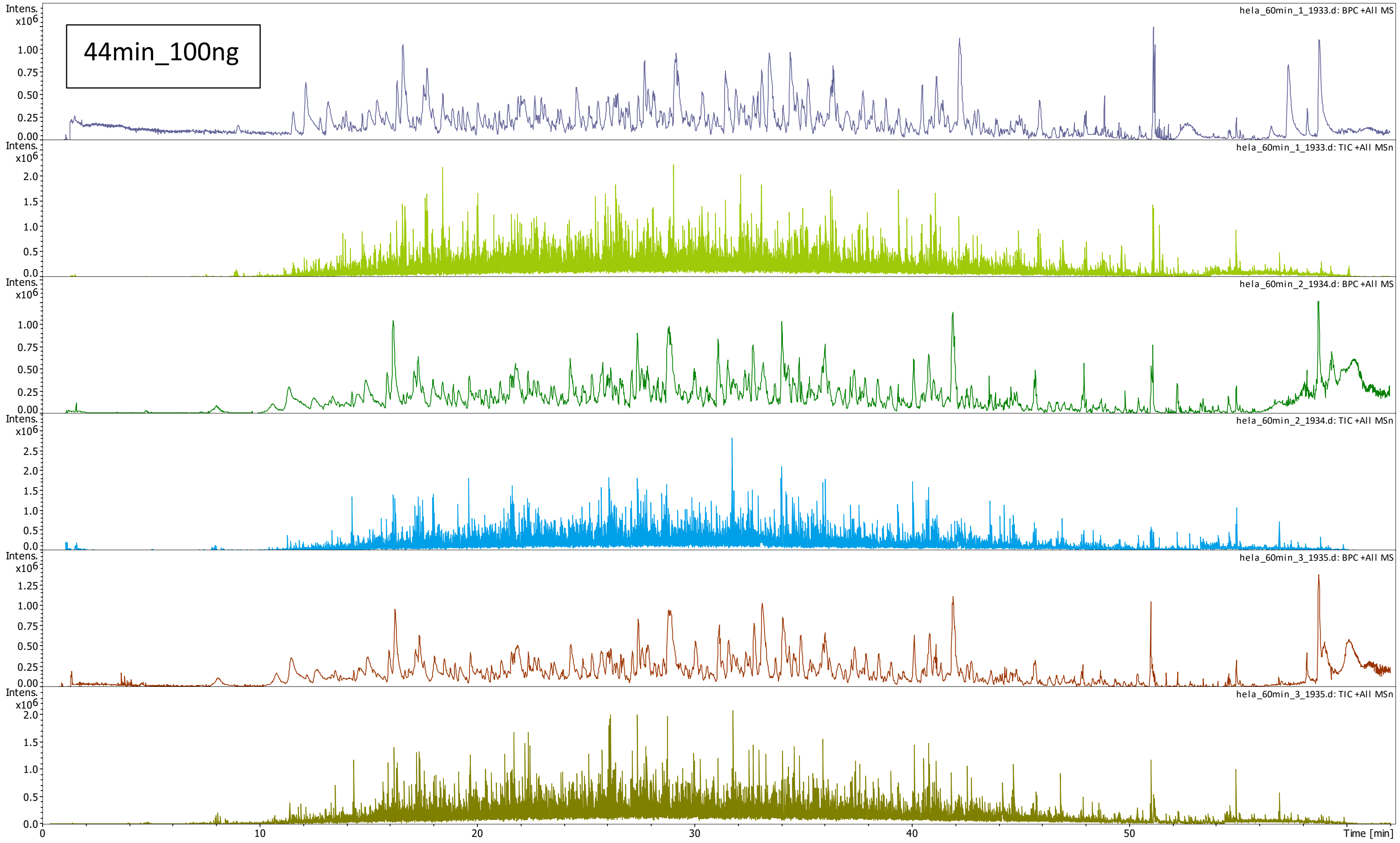

Figure S7. Base peak chromatogram and total ion current chromatogram of 44 min gradient (62 min run time) run 1 (top two), run 2 (middle two), and run 3 (bottom two) with 100 ng of HeLa digest loading.

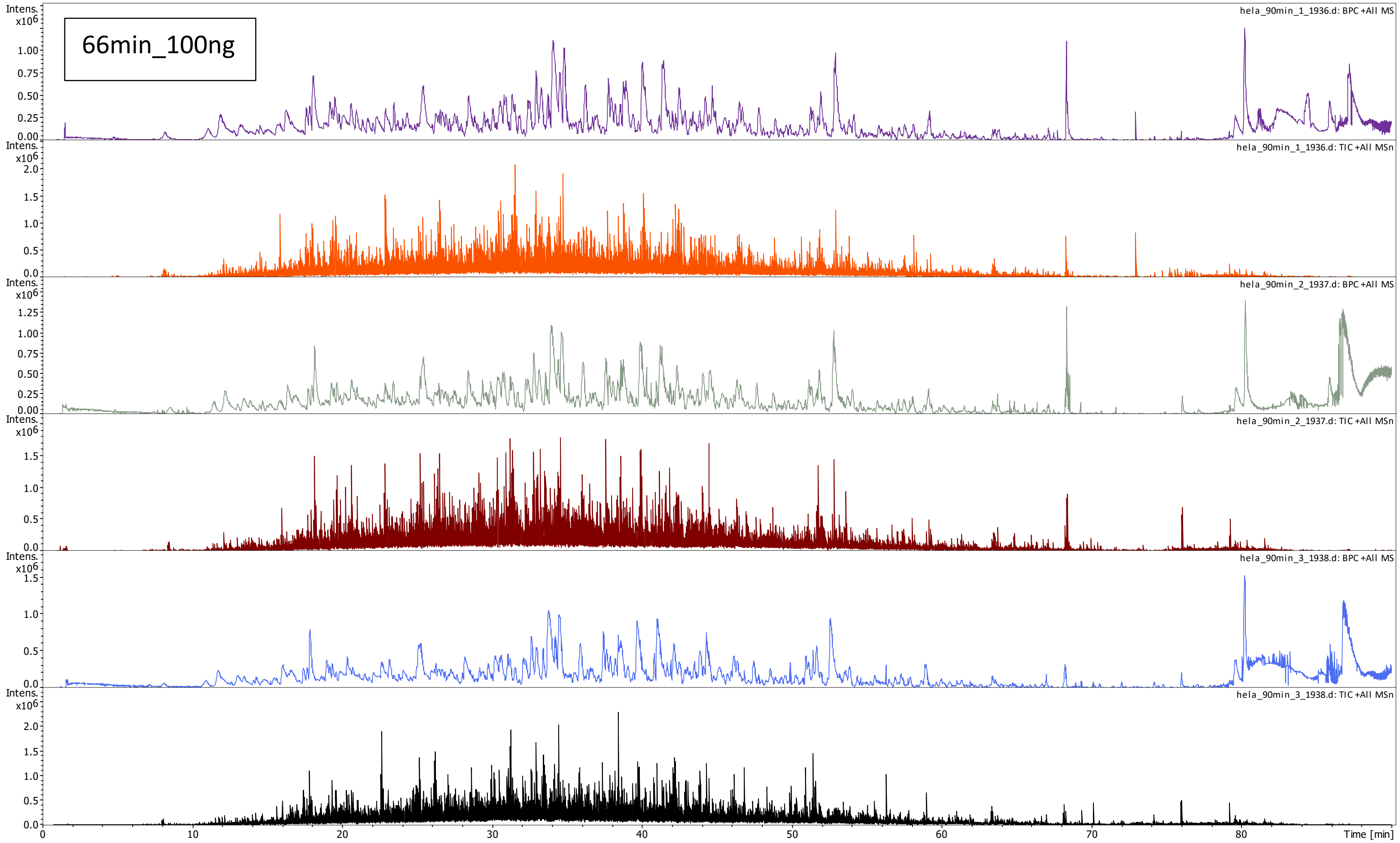

Figure S8. Base peak chromatogram and total ion current chromatogram of 66 min gradient (90 min run time) run 1 (top two), run 2 (middle two), and run 3 (bottom two) with 100 ng of HeLa digest loading.

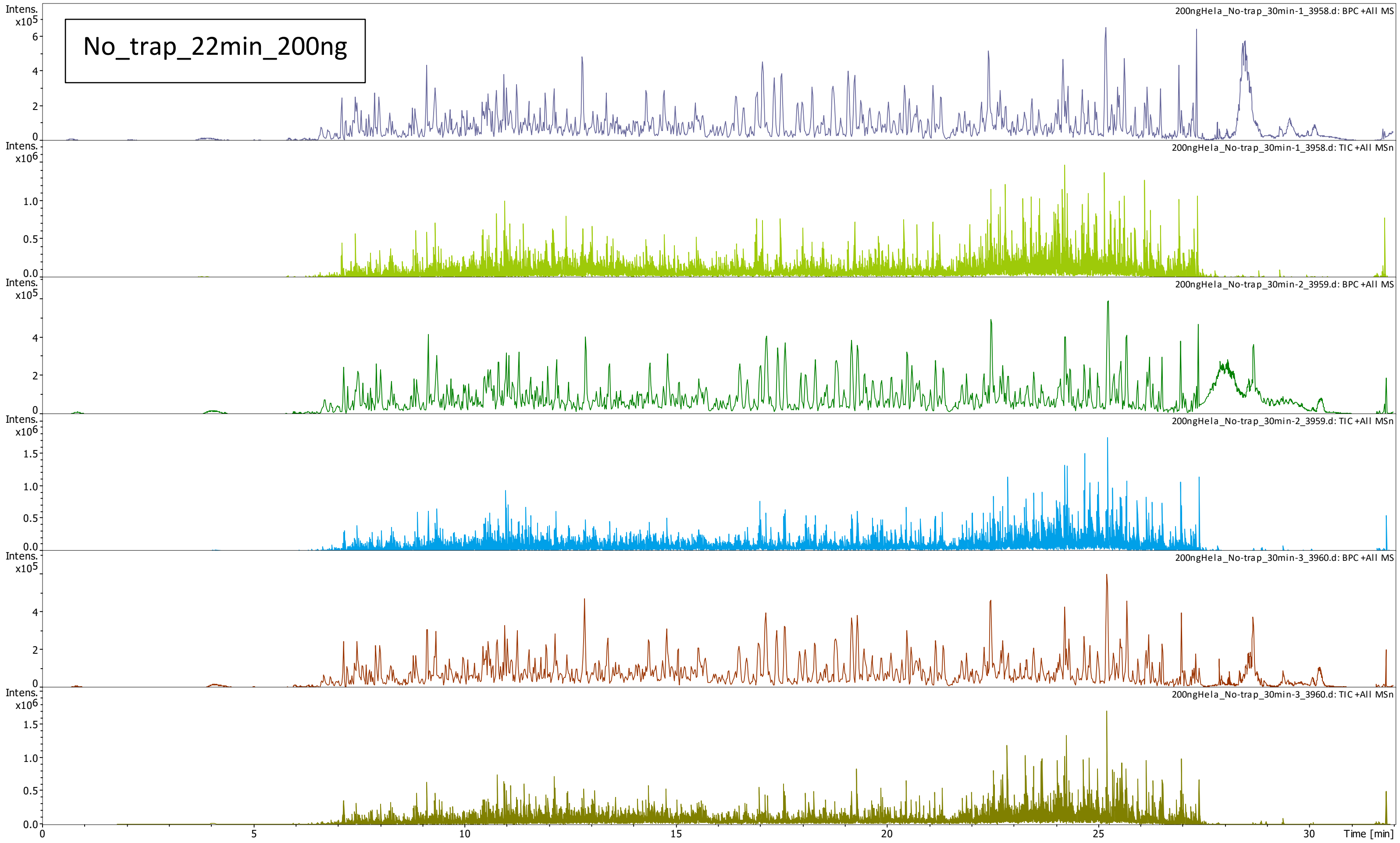

Figure S9. Base peak chromatogram and total ion current chromatogram of 22 min gradient (32 min run time) run 1 (top two), run 2 (middle two), and run 3 (bottom two) with 200 ng of HeLa digest loading without a trap column.

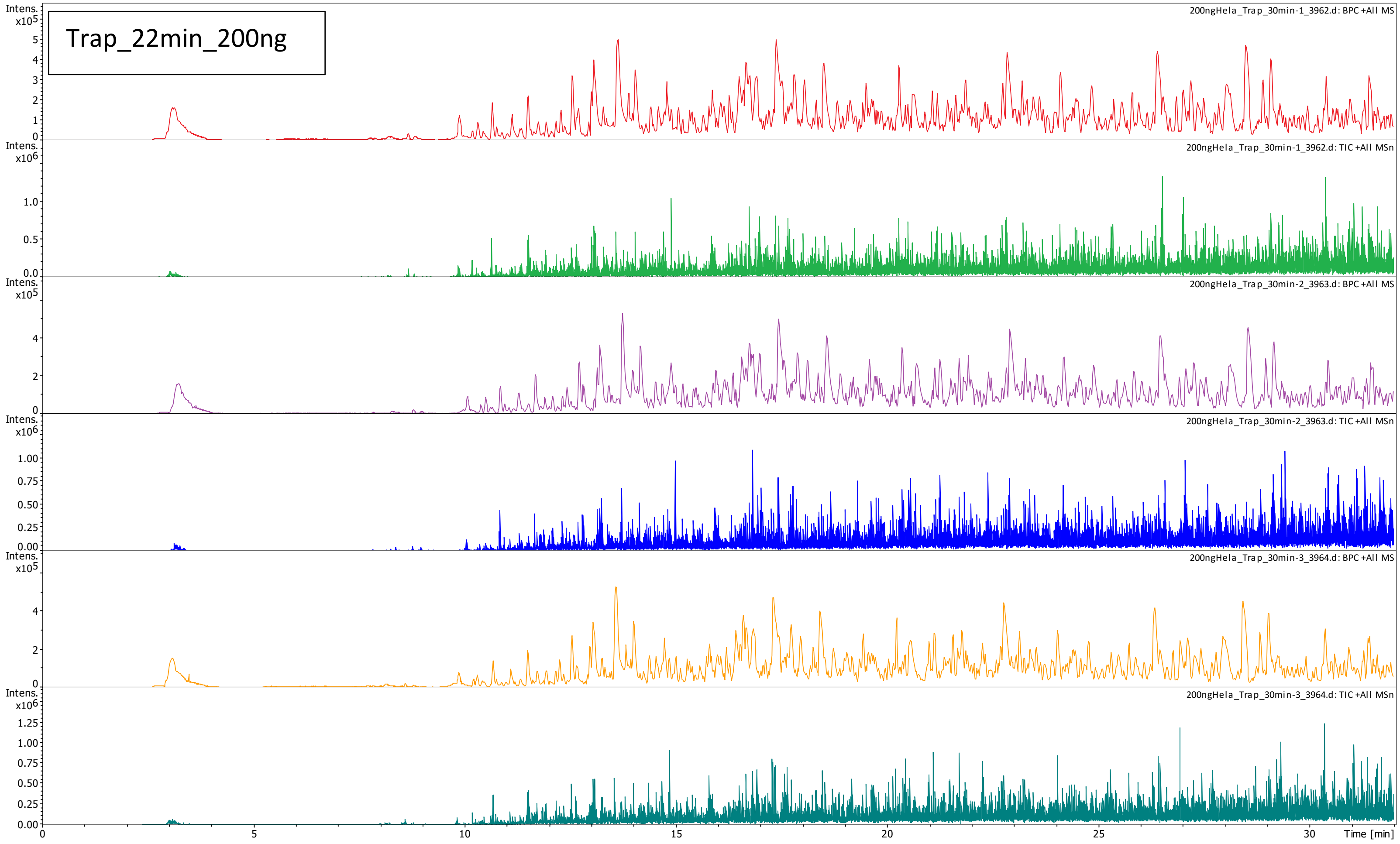

Figure S10. Base peak chromatogram and total ion current chromatogram of 22 min gradient (32 min run time) run 1 (top two), run 2 (middle two), and run 3 (bottom two) with 200 ng of HeLa digest loading with a trap column.

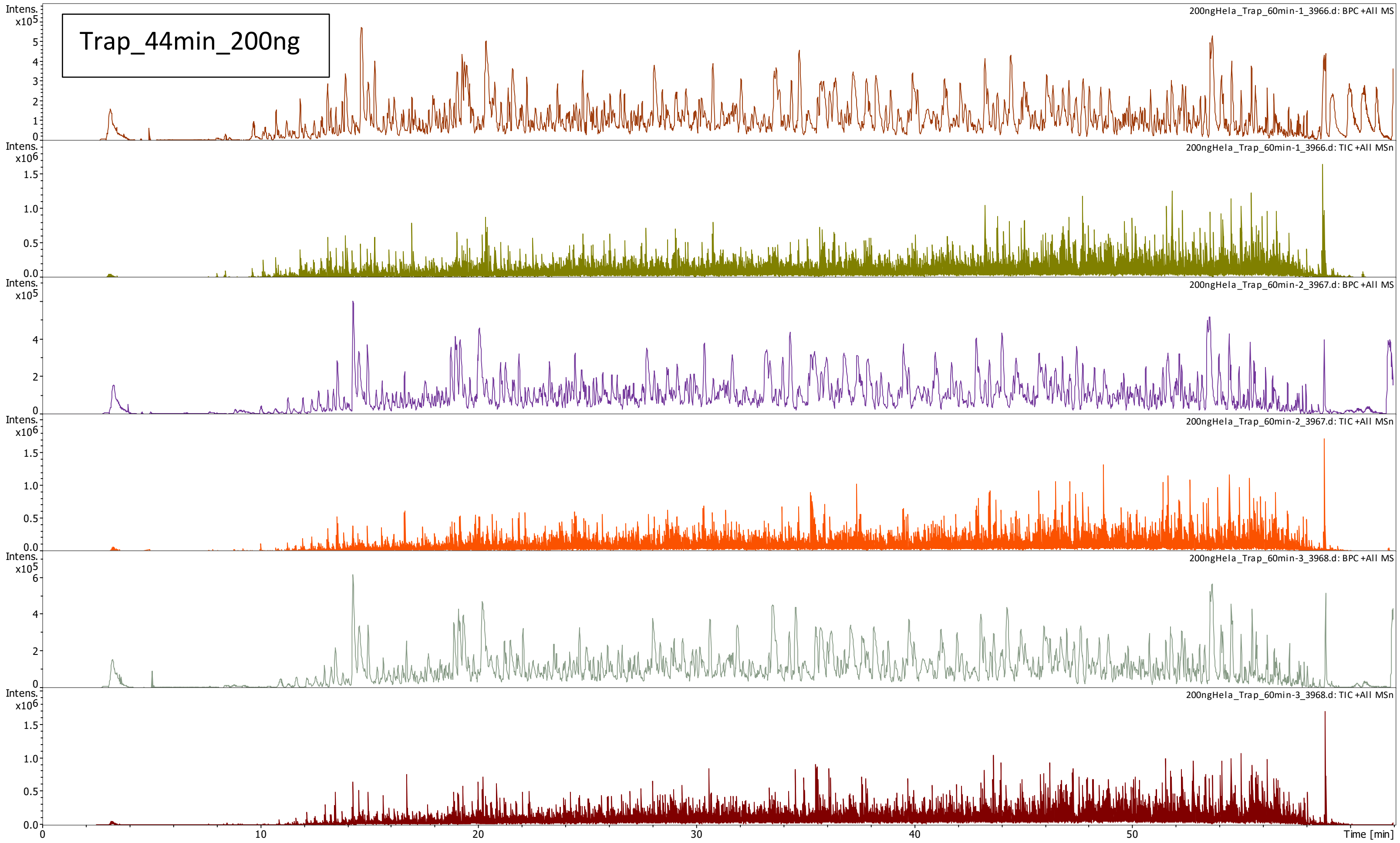

Figure S11. Base peak chromatogram and total ion current chromatogram of 44 min gradient (62 min run time) run 1 (top two), run 2 (middle two), and run 3 (bottom two) with 200 ng of HeLa digest loading with a trap column.

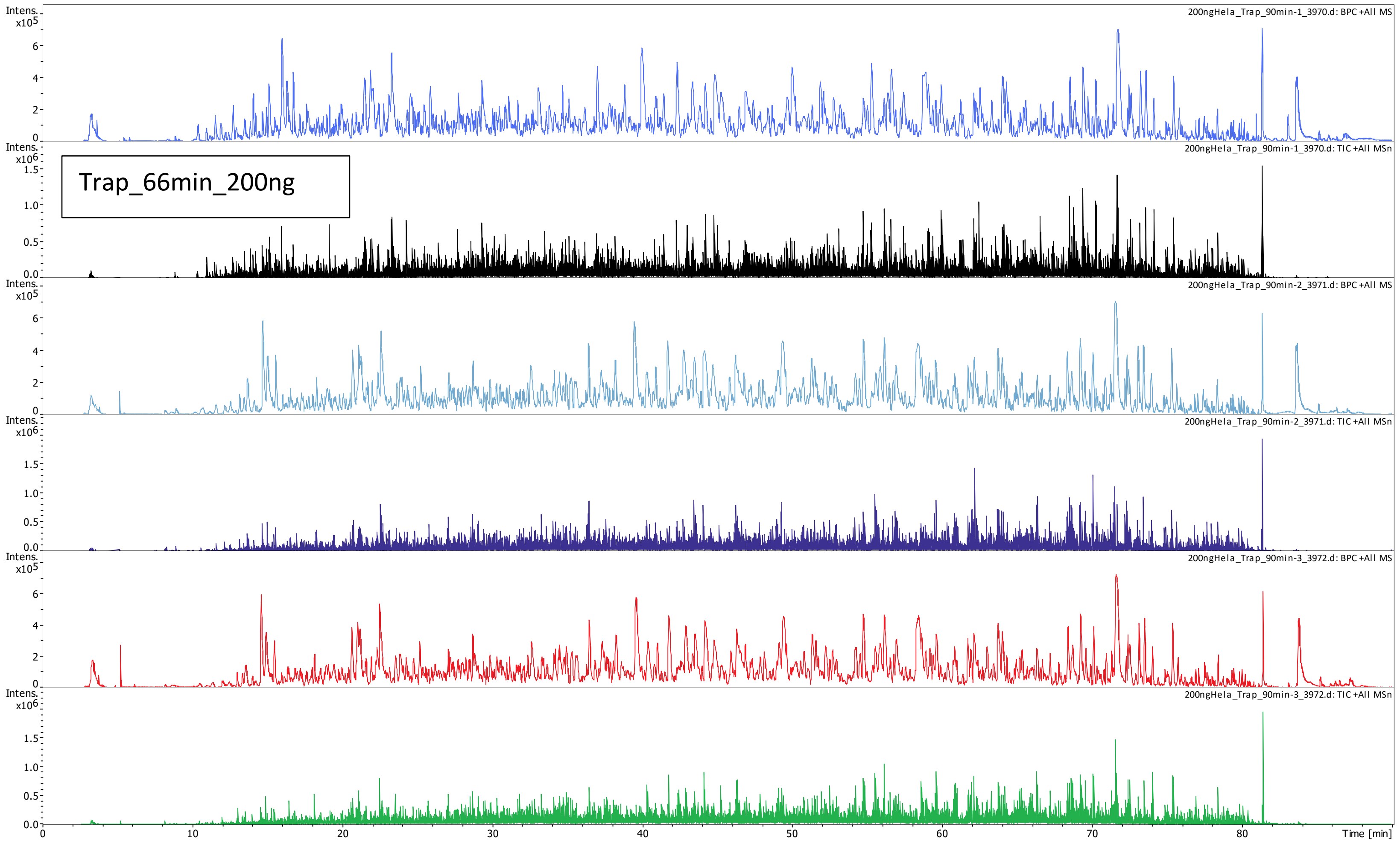

Figure S12. Base peak chromatogram and total ion current chromatogram of 66 min gradient (90 min run time) run 1 (top two), run 2 (middle two), and run 3 (bottom two) with 200 ng of HeLa digest loading with a trap column.
